# Rheological and mechanical performance of sustainable LC3 concrete modified with nano-silica and rubber latex

**DOI:** 10.1038/s41598-025-27234-z

**Published:** 2026-01-28

**Authors:** Badrinarayan Rath, T. R. Praveenkumar, Keerat Kumar Gupta, Prabhu Paramasivam, A. Jayanthiladevi, Mitiku Adare Tufa

**Affiliations:** 1 Department of Civil Engineering, Graphic Era Deemed to Be University, Dehradun, Uttarakhand India; 2 NICMAR Institute of Construction Management and Research, Delhi-NCR, Bahadurgarh, 124507 India; 3 Centre for Research Impact & Outcome, Chitkara University Institute of Engineering and Technology, Chitkara University, Rajpura, 140401 Punjab India; 4 Department of Computer Engineering, Adichunchanagiri University, Nagamangala, 571448 Karnataka India; 5 Department of Civil Engineering, Salale University, 245 Salale, Fiche, Ethiopia

**Keywords:** e-BT2 rheometer, Static yield stress, Thixotropic energy, Volumetric shrinkage, FTIR, TGA, Mechanical properties, Engineering, Civil engineering

## Abstract

Alternative sustainable and environmentally friendly cement-based materials are now possible because of substantial advancements in concrete technology. In building engineering, supplementary cementitious materials (SCMs) have gained popularity and promise as partial binder substitution in recent decades. This study examines the production of a new eco-friendly LC3 concrete (lower cement clinker concentration and CO_2_ footprint) by substituting nano silica for calcined clay. Surkhi has been used as a calcined clay in the present LC3 concrete and replaced by nano-silica at 2%, 4%, and 6% by weight. A new water replacement material has been introduced as rubber latex, which has helped to reduce the porosity of concrete. Rubber latex has replaced the water at 2% and 5%. Eight new mixes are prepared from which seven mixes are engineered LC3 concrete and one mix belongs to traditional OPC concrete of M40 grade. Several laboratory tests such as rheology, volumetric shrinkage, FTIR, TGA, compressive strength, and split tensile strength are carried out on the above-mentioned mixes of LC3 concrete and compared with the test results of traditional OPC concrete. However engineered LC3 concrete enhanced the workability by showing lower values of static yield stress, structural built-up rate and thixotropic energy as compared to traditional OPC concrete. Introducing rubber latex in LC3 concrete, the workability increases due to the production of similar charges around the cementitious materials which are repulsive. Losing the plasticity of rubber latex the voids of concrete have been filled by solid materials and correspondingly reduce the voids and shrinkage. The volumetric shrinkage was reduced by up to 84%, and compressive strength improved by 15.6% at 90 days compared to OPC concrete. Rheological tests using an eBT2 rheometer and shrinkage tests using laser measurement were performed. The mechanical and microstructural qualities of LC3 concrete have improved due to partial substitution of water with rubber latex and increased the packing density due to the partial substitution of nano-silica for surkhi.

## Introduction

Infrastructure development is mainly dependent on the cement industry, considered to be a critical component of the construction sector^1,2^. Cement production is known for its environmental impact due to high carbon dioxide emissions during the clinker manufacturing process. Clinker, the main ingredient in cement is generally produced by heating limestone and clay at a temperature of about 1400 °C^3^. During this production process large amount of carbon dioxide is released during the decomposition of limestone and combustion of fossil fuels, in addition to substantial consumption of energy^4,5^. It is estimated approximately that 7–8% of global CO_2_ emissions are mainly due to cement production and it is one of the most carbon-intensive industries worldwide^6,7^. Researchers are making several efforts to manufacture cement in a sustainable way due to growing concerns about climate change and environmental degradation. The recent approach involves the development of alternative cementitious materials that reduce clinker content while maintaining or enhancing the performance of concrete^8,9^. Limestone calcinated clay cement (LC3) emerges as a promising sustainable solution to traditional cement^10,11^. The need for sustainable alternatives in the cement industry is crucial and relying on the traditional approach of high clinker content cement results in substantial CO_2_ emissions and it requires huge energy requirements^12^. As the global demand for cement continues to increase, the environmental footprint of cement production too increases. Thus, alternative solutions including LC3 cement need to be explored to mitigate the impacts^13–15^.

LC3 is an innovative blend of limestone, calcined clay, and clinker that acts as a sustainable alternative to traditional Portland cement^16^. The significant reduction in clinker content is mainly achieved by incorporating limestone and calcined clay which reduces the carbon dioxide emissions by 30% as compared with conventional cement^17^. The replacement of clinker with calcined clay occurs at a lower temperature than that needed for the production of clinker. In addition, calcined clay can be sourced locally and it is widely available, which also reduces the raw materials dependence and promotes the local economies^18,19^. In many regions, the availability of natural pozzolanic materials including slag, and fly ash is limited which further emphasizes the importance of LC3-based concrete. The combination of limestone and calcined clay makes LC3 a more widely applicable and feasible solution in places where pozzolanic materials such as slag and fly ash are not available. The utilization of locally available materials including limestone and calcined clay significantly reduces the transportation costs and dependency of raw materials^20^. This makes LC3 a more economical option for large-scale construction projects especially in developing countries where construction costs are high. By replacing clinker partially, LC3 conserves natural resources such as limestone and clay, otherwise generally consumed in larger quantities^21,22^. Reducing the quantity of raw materials extracted reduces the environmental impact associated with quarrying. In general, quarrying involves large-scale land alterations including soil erosion and destruction of natural habitats. Reduction of limestone and clay demand and LC3 production mitigates the adverse environmental effects, making the cement manufacturing process more sustainable and environmentally friendly^23,24^. In addition, LC3 concrete shows superior performance in terms of durability, mechanical properties, and resistance to aggressive environments^25,26^.

Shi Hu et al.,^27^ explored the potential of using dehydrated concrete slurry waste (DCSW) to produce low-carbon limestone calcined clay cement through CO_2_ mineralization. The carbonated DCSW achieved a 57.7% CaCO_3_ content after 8.5 h of mineralization. Portlandite and Mc_AFm peaks diminished early during the carbonation. Replacement of limestone in Carbonated DCSW reduces the fluidity by around 15% and increases the compressive strength at all stages as compared with conventional concrete. LC3 is an advanced cementitious material that benefits from internal curing by the use of perforated cenospheres. These cenospheres can facilitate water transport through pores and it is useful in curing. LC3-based concrete significantly reduces the autogenous shrinkage by 31% and 62% when cenospheres of 4% and 8% were used respectively. Perforated cenospheres improve the microstructure and mechanical properties of LC3 concrete in addition to the reduction in shrinkage levels^28^. Eco-friendly concrete structures combining LC3 recycled concrete aggregate, seawater, sea sand, and FRP bars showed significant environmental benefits with a reduction of carbon dioxide emissions by 32% to 41.7% along with a significant energy demand decrease as compared with conventional concrete. Initial costs for LC3 concrete preparation were considerably higher but the extended lifetime and lower maintenance cost made LC3 structures economically advantageous than conventional concrete over time^29^. Marwan Abdulqader et al.^30^, assessed the feasibility of using local clays from Saudi Arabia to prepare LC3 concrete and compared them with Ukrainian clays. Three substitution levels 30%, 50%, and 70% with varying amounts of kaolinite levels in clay were analyzed for thermal and chemical properties. The incorporation of all different clays increases the water demand and setting time with a reduction in flowability values. The concrete samples prepared using these clays showed lower shrinkage and improved resistance to chloride penetration.

Nano silica, a nanomaterial consisting of extremely fine particles of silica (SiO_2_) is used by researchers these days to increase fresh and hardened concrete properties^31^. Nano silica can behave as a filler ingredient due to its pozzolanic activity and high surface area, improving the overall performance and microstructural characteristics of concrete^32–34^. In general, nano silica was added in smaller quantities to cement which ultimately improves the strength and durability. The incorporation of nano silica accelerates the hydration process of cement and leads to faster early-age strength development of concrete which is mainly helpful in the quick removal of formwork^35^. Incorporation of nano silica of different dosages from 1 to 5% improves the modulus of elasticity of concrete in addition to early age compressive strength. This is due to the reaction of nano-silica with free calcium hydroxide in concrete and produces calcium silicate hydrate gel at early stages^36^.

Recent studies have shown that combining nano and micro materials can significantly improve concrete properties. Incorporation of nano-silica (1–3%) and metakaolin (5–20%) enhanced compressive strength by up to 35% and split tensile strength by up to 27%, with optimum results at 2% nano-silica and 12.5% metakaolin at a water-binder ratio of 0.4. This synergistic effect also led to denser microstructure and higher C–S–H formation, demonstrating the potential of optimized nano and micro material dosages for sustainable high-performance concrete^46^. Nia and Shafei^47^ highlighted the potential of nano-silica as a supplementary cementitious material to enhance both fresh and hardened properties of concrete. They replaced silica fume with 1–5% nano-silica in self-consolidating concrete (SCC) showed improvements in workability, compressive and tensile strength, and durability indicators like water absorption and electrical resistivity. The optimized use of nano-silica improved the concrete’s microstructure while reducing cement demand, demonstrating its environmental and performance benefits in sustainable concrete design.

## Research significance

When cement clinker is mixed with limestone calcined clay (LC2), limestone calcined clay concrete (LC3) is produced. Cement hydration is improved in LC3 concrete by the mixing of the above ingredients because limestone serves as filler and provides enough surface area for hydrates. Portal water is an essential ingredient for the preparation of LC3 concrete by mixing it with a dry mix. Cementitious materials of concrete react with water and produce CSH gel. Those CSH gel seals the capillary pores developed inside the concrete and reduces the void gap of the concrete, by which the mechanical properties and durable qualities of the concrete increases. Many researchers are searching for an alternative material to replace the water to reduce the porosity of concrete in a better manner. A limited study has been carried out regarding water replacement material for the production of concrete. Rubber latex is utilized in the current study as a water substitute, which works better as a pore sealant. The unit weight of rubber latex is similar to the unit weight of the water. Hence, rubber latex mixes with water thoroughly and produces a homogenous concrete mix without any trouble. As the time passes, the liquid rubber latex loses its plasticity and becomes solid. Those solid materials help to plug the porous nature developed inside the mix sample of concrete and reduce the porosity of concrete. Therefore the rubber latex has been selected as a water replacement for the present study.

In this study, LC3 concrete was prepared by a combination of limestone powder, surkhi, and OPC cement clinker. In the previous studies, the cement clinker had been carried as 50% by weight of the total mix. To reduce the use of cement clinker in LC3 concrete, nano silica had been introduced to replace calcined clay partially up to 6% and the amount of cement clinker had been restricted up to 33%. Nano silica was selected for the present study because it contained a maximum percentage of silica and played a crucial role in the hydration process. Water was substituted partially by rubber latex up to 5%. The combined solution of water and rubber latex had been added to the dry mix of nano silica-based LC3 concrete and a new type of homogenous mixed LC3 concrete had been prepared. By the combination of nano silica and rubber latex, seven new types of engineered LC3 concrete had been produced in the laboratory. Several parametric studies such as rheology, volumetric shrinkage, mechanical properties, and microstructural properties have been studied and compared with traditional OPC concrete.

The novelties of the present studies are as follows.

(i) Limited research has been carried out for alternative water replacement material for the preparation of the LC3 concrete.
(ii) Limited research has been studied regarding several rheological parameters such as static yield stress, structural built-up rate, thixotropic energy, etc. on LC3 concrete.
(iii) Limited research has been studied regarding volumetric shrinkage and corresponding strains of LC3 concrete. Most of the researches are related to linear shrinkage of LC3 concrete.
(iv) Limited research has been done on Thermo Gravimetric Analysis of LC3 concrete.
(v) An engineered LC3 concrete has been prepared to restrict the use of cement clinker up to 33%.

## Materials used and their mix proportions

**Fig. 1:**
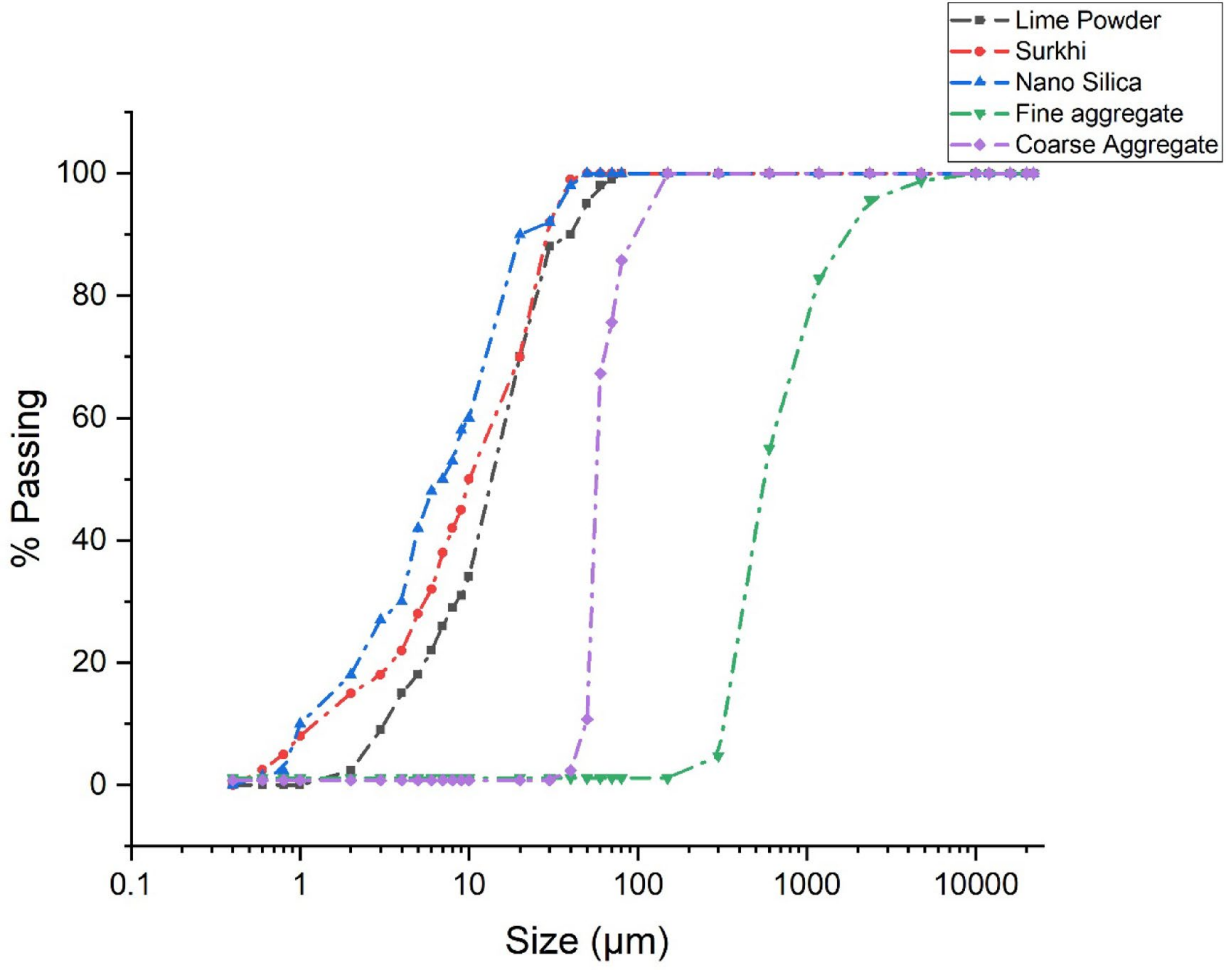
Particle size distribution curves of lime powder, surkhi, nano silica, fine aggregate and coarse aggregate.

Limestone calcined clay cement (LC3) is a promising new technology that is made by substituting clinker with calcined clays and limestone powder to create a tertiary blend. Hence in the present investigation, limestone powder, surkhi, nano silica, and cement were taken as the binder materials to prepare a new type of engineered cement concrete. The surkhi was obtained from the waste calcined broken brick pieces collected from the bottom of traditional brick kilns in India, where bricks are typically made using alluvial soil rich in silica and alumina—common in regions such as Uttar Pradesh. These soils, derived from the Indo-Gangetic plains, make the fired bricks suitable for pozzolanic applications. The calcination temperature for producing clay brick typically ranges between 600 and 800 °C, with an optimal duration of 4 to 6 h, although the total residence time in traditional kilns may extend up to 48 h. To maintain optimum pozzolanic reactivity, brick overburning (above 950 °C) is avoided, as excessive heating can lead to glassy phase formation and loss of amorphous silica reactivity. The broken fire red bricks collected from the bottom of the kiln. The calcined brick waste was then pounded in a ball mill and sieved to obtain a fine powder, ideally passing through a 75 µm sieve, with a target specific surface area of at least 300 m^2^/kg to ensure adequate fineness for cementitious use. Since the surkhi (i.e. calcined clay) possesses 78.8 weight percent of amorphous content, according to the XRD-Rietveld study^37^, it was used in this study for making high-grade calcined clay. In an earlier study, the authors observed that the mineral composition of calcined clay included dickite, anatase, kaolinite, mullite, and quartz^38^. The limestone powder, which contained more than 95 percent of CaCO_3_, was purchased from the local market (Sigma Minerals Ltd., Jodhpur, India). According to the company’s brochure, the CaCO₃ purity is stated to be greater than 95%. The commercially available spherical nano-silica (70–85 nm in diameter, 99.9% purity, BET surface area ~ 200 m^2^/g) sourced from Astra Chemicals was used as a partial substitution for calcined clay in a range of proportions from 1 to 6 percent with a 2 percent increment. Nano-silica acted primarily as a pozzolanic filler, densifying the matrix, whereas rubber latex served as a pore-sealing agent and partial water replacement, enhancing workability and shrinkage control. The concrete mixing process employed a homogenizing technique to enhance the dispersion of nanoparticles. Table 1 summarizes the chemical composition of limestone powder, calcined clay, and nano-silica which are determined by X-ray fluorescence. The laser diffraction method was used to measure the size distribution of their particles as shown in Fig. 1. In the current study, the largest diameters of the crushed coarse and fine aggregates were 20 mm and 4.75 mm, respectively. A coarse aggregate encraved from basalt stone with a maximum nominal size of 20 mm and water absorption of 1.08% was utilized. Locally available sand of size below 4.75 mm having a water absorption capacity of below 3.5% was utilized. To eliminate any moisture prior to concrete casting, every aggregate was oven-dried for a minimum of twenty-four hours at 105 °C in order to attain a consistent mass.

**Table 1:** Chemical composition of lime powder, Surkhi and Nano Silica.

Composition	OPC	Lime powder	Surkhi	Nanosilica
CaO	63.52	56.87	0.18	0.15
SiO_2_	21.67	1.2	50.33	98.1
Al_2_O_3_	5.24	0.28	41.21	0.08
Fe_2_O_3_	4.13	0.18	3.1	0.005
MgO	1.18	1.53	0.08	0.16
SO_3_	2.18	0.04	0.09	0.02
Na_2_O	0.2	0.04	0.35	0.07
K_2_O	0.45	0.01	0.08	0.06
TiO_2_	–	–	2.3	0.04
Loss of ignition (LOI)	1.43	39.85	2.28	1.315

The effects of calcined clay, nano silica, and limestone powder (LC3) on the mechanical characteristics and shrinkage development of concrete were examined by creating eight different concrete mixtures of M40 grade. Concretes with merely ordinary Portland cement and those with ordinary Portland cement, limestone, calcined clay, and nano-silica as a binder were indicated by CM and LC3, respectively. The percentage of OPC in the LC3 binder was found as almost 50% of the overall binder weight, in earlier research^39,40^. But to reduce the consumption of the cement in actual practice, it was decided that to restrict the use of cement in present LC3 up to 40%, the three binders i.e. OPC, limestone powder, and calcined clay were used in equal percentages i.e. 33%. To increase the packing density of LC3 concrete nano-silica was used to replace the calcined clay up to 6%. Rubber latex has been used in part as a substitute for water in the current study since both are available in liquid form. In addition, natural rubber latex has a unit weight that is similar to that of the water and flowing freely. The IS-10262:2019^41^ states that the predicted water content of concrete (as determined from mix proportion method) can be decreased upto 10% when superplasticizers are added at the correct quantity. It has been discovered that adding rubber latex to geopolymer concrete improves its rheology and workability characteristics^42^. Hence it had been decided to replace the water with rubber latex at 2% and 5%. A consistent concrete volume and total binder content were used in the formulation of each mix. The same concrete grade also had fixed water/binder and coarse/fine aggregate volumetric ratios. Prior to concrete casting, both coarse and fine aggregates were cooled after oven drying, and the SSD condition was achieved by adding the appropriate amount of SSD water. Cooling aggregates and achieving SSD conditions minimize variations in surface moisture and heat, which could otherwise affect workability, early hydration kinetics, and strength development. To achieve suitable workability, a super plasticizer (0.5% of binder content) was used during mixing. The mix composition of the proposed concrete is shown in Table 2. Fresh concrete was mixed, then poured into various molds and sealed with the proper lids to stop moisture from evaporating from the surface. The process of casting concrete took place in a regulated environment with a temperature of 25 ± 2 °C and a relative humidity of 50 ± 4%. After a day, the hardened concrete was de-molded and various curing and pre-conditioning regimens that complied with the various standard protocols detailed in the following section were followed.

**Table 2:** Details on the mix composition of concretes.

Materials (kg/m^3^)	CM	LC_0–0_	LC_2–2_	LC_2–5_	LC_4–2_	LC_4–5_	LC_6–2_	LC_6–5_
OPC	413	137.6	137.6	137.6	137.6	137.6	137.6	137.6
Lime stone powder	0	137.6	137.6	137.6	137.6	137.6	137.6	137.6
Calcined clay	0	137.6	134.84	134.84	132.1	132.1	129.34	129.34
Nano silica	0	0	2.75	2.75	5.5	5.5	8.25	8.25
Fine aggregate	640	640	640	640	640	640	640	640
Coarse aggregate	1225	1225	1225	1225	1225	1225	1225	1225
Water	166	166	162.68	157.7	162.68	157.7	162.68	157.7
Rubber latex	0	0	3.32	8.3	3.32	8.3	3.32	8.3
Super placticizer	2.06	2.06	2.06	2.06	2.06	2.06	2.06	2.06

## Experimental programs

### Rheological properties

**Fig. 2:**
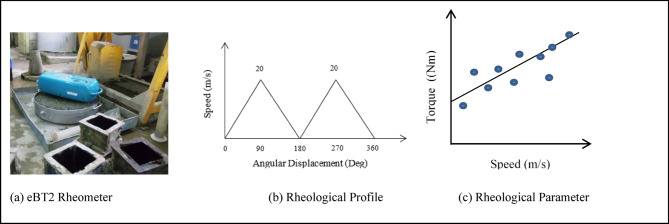
Rheology of different concrete specimen.

In the current study, LC3 concrete had been used by introducing nano silica replacing calcined clay up to 6% by weight for improving the rheological properties. For observing the rheological properties e-BT2 type of rheometer was used and the relative viscosity and yield stress of each mix were measured as shown in Fig. 2a. The rotating motion theory underpins the operation of the eBT2 rheometer. Applying torque or coupling can control stress and quantify the rate at which strain is produced. The system consists of two legs similar to probe positioned on opposite faces of a central axis, spaced 43 cm apart. These probes are arranged asymmetrically, with one positioned closer to the shaft and the other farther away. This design ensures that the probes follow different paths during rotation, resulting in distinct angular speeds for each probe. As a consequence, two torques could not be measured at the same location, preventing any separation of aggregate from the cement mortar within the LC3 concrete sample. The average of the two values was recorded by the rheometer.

Approximately nineteen liters of desired mix of concrete were prepared and filled into a cylindrical container. The shaft of the rheometer was placed in the container’s center slot. The rotational speed of the e-BT2 typed rheometer varied, with a maximum speed of 20°/sec. During the first quarter of the 180° rotation, there was an increasing of angular velocity from 0°/sec to 20°/sec, and during the second phase of quarter, it gradually decreased back to 0°/sec. In this way, it repeated for the next two quarters as shown in Fig. 2b. Measurements of several rheological parameters such as static yield stresses, dynamic yield stress and relative viscosity were taken for all eight mix types. The entire process was remotely controlled via a smartphone, and upon completion, all readings are transferred to the device via Bluetooth. The results were then displayed graphically on the smartphone screen as shown in Fig. 2c. The slope of speed vs torque curve revealed the relative viscosity of the concrete mix, while the Y-intercept had shown the yield stress. The linear part of the down curve was extended to the Y-axis (i.e. Y-intercept) in order to calculate the dynamic yield stress for various combinations which was captured on a smartphone is shown in Fig. 2c.

From the above data Static Yield Stress, Thixotropic Energy and Structural Built-up Rate were evaluated for different mixes of LC3 concrete.

### Shrinkage test

**Fig. 3:**
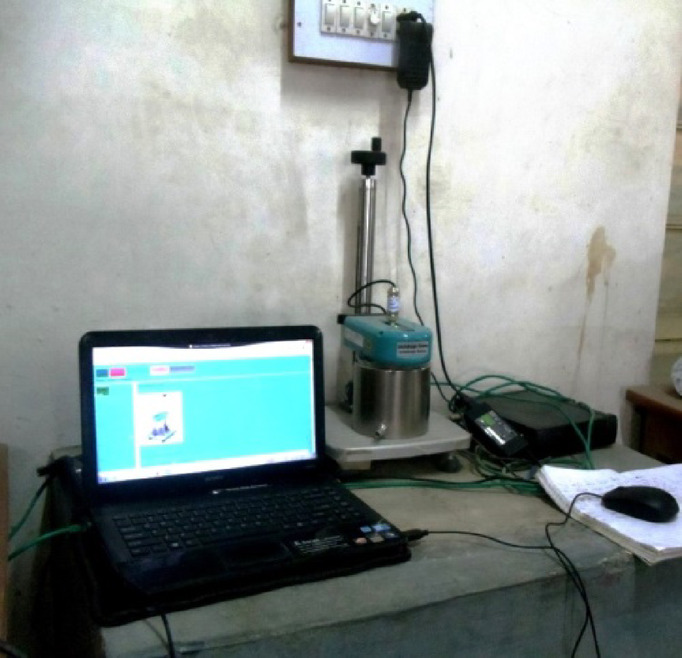
Shrinkage test set up for several mixes.

The shrinkage of eight mixes throughout their volume (i.e. volumetric shrinkage) was observed by using a shrinkage meter, as illustrated in Fig. 3. In this research, a mold with cone shape was used to determine the volumetric shrinkage. Previous studies often measured shrinkage using rectangular prism-shaped concrete beams, focusing on changes in length over time. However, since the prism of rectangular in shape shrinks in three dimensions but measurements were only taken in the longitudinal direction; those results reflected only shrinkage of linear type, not the actual shrinkage throughout its volume. In contrast, this study used a conical mold to capture true shrinkage throughout its volume (i.e. volumetric shrinkage).

When shrinkage of conical shape of concrete takes place, the cone’s height and radius both drop proportionately and concurrently, indicating that the volume loss and height change is intimately correlated. By measuring the reduction in the cone’s height, the volume reduction could be calculated. As noted from the previous research^43^, the shrinkage process shows that the decrease in the cone’s height is directly proportional to the reduction in its volume.

After mixing, fresh concrete was poured into a conical mold with a diameter of 11 cm and a height of 9 cm. A laser beam was continuously focused on the top of the conical specimen. As time passed, the fresh concrete shrank and gradually settled, causing a reduction in its initial height. This height reduction was detected by the laser at 15-min intervals over eight hours. The shrinkage results for different concrete mixes were determined.

### Mechanical properties

In order to evaluate the mechanical properties, such as compressive strength and split tensile strength, standard concrete cubes measuring 150 mm × 150 mm × 150 mm in size and cylinders measuring 300 mm in height and 150 mm in diameter respectively were employed. Those specimens were continually cured in fresh water for up to 90 days at 25 ± 2 °C. After casting, compressive strength and split tensile strength were measured for both OPC concrete and LC3 concrete at 28 days and 90 days of water curing.

### Microstructural properties

#### FTIR test

Once the mechanical test was completed, a tiny section of the specimen was removed from the fractured area and immediately saturated in acetone to prevent additional hydration. For FTIR characterization, it was thereafter finely powdered. After mixing of the specimen with KBr, measurements were made using total attenuated reflection mode in the frequency range of 500–4000 cm^–1^. Using Fourier transform infrared spectroscopy (FTIR) and a Bruker ALPHA spectrophotometer, the specimens were examined for structural alterations. Once the mechanical test was completed, a tiny section of the specimen was removed from the fractured area and immediately saturated in acetone to prevent additional hydration. For FTIR characterization, it was thereafter finely powdered. After mixing the specimens with KBr, total attenuated reflection mode measurements were performed in the frequency range of 500–4000 cm^–1^. Fourier transform infrared spectroscopy (FTIR) using a Bruker ALPHA spectrophotometer was used to examine the specimens for structural alterations.

#### TGA analysis

Following a 28-day curing period, each concrete specimen mix was crushed into a powder and heated to approximately 800 °C at a consistent rate of 10 °C/min. A thermo-gravimetric device was utilized to estimate the mass fraction of C–S–H and their corresponding hydration products of various mixtures. The mass loss between temperatures 520 °C and 750 °C was used to calculate the amount of calcium carbonate in ordinary Portland cement concrete and LC3 concrete. For all kinds of mixtures, the temperature range was discovered on the observations of the peak owing to mass loss. In addition, the ratio of calcium that reacted during the carbonation process at lab practice to the theoretical quantity was determined using the TGA findings to determine the reaction capability of calcium during the process.

## Result and discussion

### Rheology test

It is frequently challenging to precisely characterize the flow characteristics of any concrete prepared from several binding materials since the different approved testing conditions, such as force application, sliding of two layers of concrete layer, and equipment state of evaluation, are qualitative in nature. These qualitative tests, which include numerous variables that are very challenging to maintain as constants, include the compaction factor test, slump cone test, flow table test, L-Box test, and others. In order to establish for the purpose to offer a more scientific application and understand the impacts of nano silica and rubber latex on both OPC concrete and LC3 concrete, rheological information was therefore determined. Additionally, it offers an exact quantitative assessment of the workability of several mixes of concrete. It was observed that the rheological parametric values of LC3 concrete were higher than the traditional concrete mix. The static yield stress, dynamic yield stress and relative viscosity for LC3 concrete had decreased 3.2%, 5.8%, and 18.6% respectively. It happened due to the finer particles of limestone powder and calcined clay. It is noticed that when the calcined clay was replaced with nano silica up to 6%, the rheological parameters like yield stress gradually decreased. This happened because the tiny, spherical nano-silica particles acted as lubricants by creating a ball-bearing effect. Since nano silica particles contributed less heat of hydration, concrete had less water loss. Thus, it may be said that workability was improved by replacing 6% of the calcined clay with nano silica, partially by weight. In comparison to the introduction of nano-silica, both the yield stress and viscosity of concrete mixes decreased rapidly when 5% of the water was substituted with rubber latex. The liquid rubber latex formed a film around each particle of cement, stone powder, and calcined clay, imparting a similar charge to each one. This uniform charge created a repulsive force between the particles, causing them to disperse more easily. As a result, the mobility of the concrete mix at fresh stage increased, facilitated by the partial substitution of water with rubber latex.

**Fig. 4:**
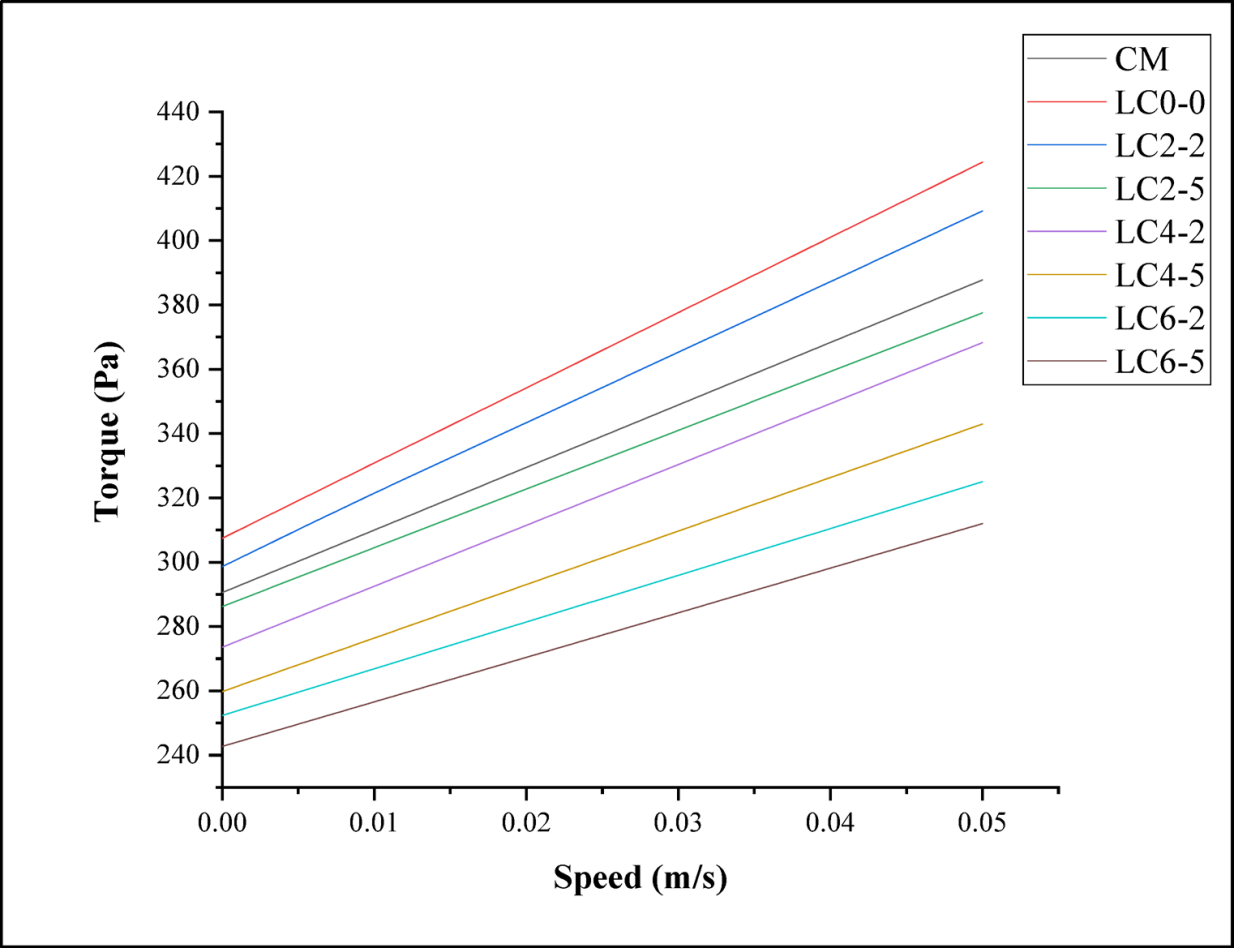
Torque vs speed curve for LC3 concrete mixes.

The Torque vs Speed relationship for all mixes exhibited a nearly linear trend, which confirmed that the flow behavior of both the control and LC3 concretes followed the Bingham plastic model. In this model, torque (or shear stress) initially resisted motion due to the presence of a finite yield stress (τ₀), and beyond this threshold, the material flowed proportionally with increasing shear rate (or speed) governed by the plastic viscosity (μ) as shown in Fig. 4. The slope of each line in the plot represents the viscous component of the flow, while the intercept on the torque axis corresponds to the yield stress. Among all mixes, LC_0–0_ and LC_2–2_ exhibited the highest torque values across the speed range, indicating higher yield stress and viscosity. This behavior could be attributed to the synergistic effect of limestone powder and calcined clay, which enhanced particle packing density and interparticle friction, leading to the formation of a stronger flocculated microstructure. The presence of fine calcined clay particles increased water adsorption and interparticle cohesion through pozzolanic and van der Waals interactions, thereby increasing internal resistance to flow.

In contrast, the LC_6–5_ and LC_6–2_ mixes show the lowest torque at comparable speeds, which was indicative of reduced yield stress and viscosity. The higher dosage of nano-silica (6%) and rubber latex (5%) acted as a dispersing and lubricating agent, respectively. Nano-silica improves particle dispersion due to its high surface charge, while rubber latex introduced a polymeric film around the particles, reducing internal friction and flocculation. Consequently, the mixes became more flowable with lower energy requirements for shearing, which was reflected in the reduced torque response. The intermediate mixes such as LC_2–5_ and LC_4–2_ exhibited moderate torque values, suggesting a balance between cohesion and workability. The progressive decreased in both torque magnitude and slope from LC_0–0_ to LC_6–5_ highlighted the rheological transition from a cohesive to a more fluid system as nano-silica and polymer content increased. Scientifically, this trend was linked to the competition between particle agglomeration and dispersion:

- At lower nano-silica content (2–4%), flocculation dominated due to limited surface modification, leading to higher structural build-up.
- At higher nano-silica and latex levels, dispersion effected surpass flocculation, resulting in reduced yield stress and viscosity.

Overall, the observed variations reflected the influence of LC3 binder composition on the microstructural evolution of cement paste during shear. The torque–speed curves validated that limestone–calcined clay blends possess inherently higher structural integrity than the control mix, but the addition of excessive dispersing components (nano-silica and polymer) could lead to over-dispersion, reducing cohesive strength. Hence, optimum rheological behavior—characterized by moderate torque, high thixotropic recovery, and stable flow—was achieved for intermediate combinations such as LC_2–2_ and LC_4–2_, which provided the best compromise between workability and stability.

The rheological parameters of the control mix and seven LC3 concretes were further utilized to estimate the Thixotropic Energy (Eₜₕ) and Structural Build-Up Rate (Aₜ), which describe the reversible structure formation and static yield stress evolution in cementitious systems. The Bingham model, expressed as

1 $$\tau = \tau_{0} + \mu \dot{\gamma }$$

was employed to represent the flow behavior, where $\tau$ is the shear stress (Pa), $\tau_{0}$ is the yield stress (Pa), $\mu$ is the plastic viscosity (Pa·s), and $\dot{\gamma }$ is the shear rate (s⁻^1^). Thixotropic energy $E_{th}$ has been defined as the area enclosed between the up- and down-ramp flow curves (hysteresis loop) measured over the shear-rate interval $\left[ {\dot{\gamma }_{min} , \dot{\gamma }_{max} } \right]$, following standard rheological practice for cementitious materials. When each ramp is well described by Bingham fits $\tau = \tau_{0}{up} + \mu_{up} \dot{\gamma }$ and $\tau = \tau_{0}{down} + \mu_{down} \dot{\gamma }$, the enclosed area admits an analytical expression as the integral of the difference of the two lines across the interval.

2 $${\text{Mathematically }}E_{{th}} = \int\limits_{{\dot{\gamma }_{{min}} }}^{{\dot{\gamma }_{{max}} }} {\left( {\tau _{{0\;up}} - \tau _{{0\;down}} } \right)\dot{\gamma }}$$

3 $$\Delta \tau_{0} = \left( {\tau_{0\;up} - \tau_{0\;down} } \right) + \left( {\mu_{up} - \mu_{down} } \right)\;\dot{\gamma }$$

4 $$Hence,\Delta \tau = \Delta \tau_{0} + \Delta \dot{\gamma }$$

Now substituting $\Delta \tau$ into the thixotropic energy integral.

4 $$E_{th} = \mathop \smallint \limits_{{\dot{\gamma }_{min} }}^{{\dot{\gamma }_{max} }} \left( {\Delta \tau_{0} + \Delta \dot{\gamma }} \right){\mathrm{d}}\dot{\gamma }$$

Assuming the test starts from rest (i.e. $\dot{\gamma }_{min} = 0$) and ends at $\dot{\gamma }_{max} = \Delta \dot{\gamma }$.

To quantify the energy associated with the breakdown and rebuilding of the microstructure, the Thixotropic Energy (Eₜₕ) was estimated from the area enclosed between the up-ramp and down-ramp flow curves as follows.

6 $$E_{th} = \mathop \smallint \limits_{0}^{{\Delta \dot{\gamma }}} \left( {\Delta \tau_{0} + \Delta \dot{\gamma }} \right){\mathrm{d}}\dot{\gamma }$$

7 $$E_{th} = \left( {\Delta \tau_{0} } \right)\Delta \mathop {\gamma + }\limits \frac{1}{2}\left( {\Delta \mu } \right)\left( {\Delta \dot{\gamma }} \right)^{2}$$

where $\Delta \tau_{0}$ and $\Delta \mu$ represent the difference in yield stress and viscosity between the up-ramp and down-ramp curves, respectively, and $\Delta \dot{\gamma }$ is the shear-rate range during testing.

Based on previous rheological studies of cement-based materials, a typical shear-rate range of 0–5 s⁻^1^ was considered. The down-ramp yield stress and viscosity were assumed to be 15% lower than their respective up-ramp values to account for partial structural breakdown during shear. Substituting these parameters for each mix allowed the computation of $E_{th}$ in Pa·s⁻^1^. The calculated thixotropic energy varied between 1.69 × 10^5^ and 2.68 × 10^5^ Pa·s⁻^1^, indicating that all LC3 concretes exhibited measurable time-dependent behavior.

The evolution of static yield stress with rest time was used to estimate the Structural Build-Up Rate ($A_{t}$) according to.

8 $$\tau_{0, static} \left( t \right) = \tau_{0} + A_{t} t$$

where $\tau_{0, static}$ is the static yield stress after a resting period t (min), $\tau_{0,0}$ is the initial yield stress immediately after mixing, and $A_{t}$ (Pa/min) is the slope representing the rate of structure formation. Since direct time-dependent data were not available, $A_{t}$ was estimated using a proportional relationship derived from the ratio of yield stress and viscosity, as these parameters are known to strongly influence early structuration kinetics. The calculated $A_{t}$ values ranged between 0.33 and 0.73 Pa/min, signifying moderate to strong structural build-up characteristics typical of limestone–calcined clay systems.

The results demonstrated a consistent trend where both Eₜₕ and Aₜ decreased with increasing nano-silica and rubber-latex contents and quoted in Table 3. The LC_0–0_ and LC_2–2_ mixes exhibited the highest thixotropic energy (2.68 × 10^5^ and 2.49 × 10^5^ Pa·s⁻^1^, respectively) and build-up rates (0.73 and 0.64 Pa/min), reflecting a stronger flocculated network facilitated by the synergistic interaction between calcined clay and limestone powder. The control mix (CM) showed intermediate thixotropy (2.35 × 10^5^ Pa·s⁻^1^) and build-up rate (0.58 Pa/min), while mixes containing higher nano-silica and latex levels (LC_4–5_, LC_6–2_, and LC_6–5_) demonstrated progressively lower values, indicating a more dispersed particle structure and improved flowability. The lowest Eₜₕ (1.69 × 10^5^ Pa·s⁻^1^) and $A_{t}$ (0.33 Pa/min) were recorded for LC_6–5_, confirming that excessive nano-silica and latex reduce interparticle friction and hinder structural regeneration.

**Table 3:** Rheological and thixotropic parameters of control and LC3 concrete mixes derived from Bingham model analysis.

Mix	τ₀ (Pa)	μ (Pa·s)	Δτ₀ (Pa)	Δμ (Pa·s)	Eₜₕ (Pa·s⁻^1^)	Aₜ (Pa/min)	Interpretation
CM	290.45	19.96	43.57	3	235.6 × 10^3^	0.58	Moderate thixotropy; balanced structuration
LC_0–0_	307.45	23.68	46.12	3.55	268.3 × 10^3^	0.73	Highest thixotropy; limestone–clay synergy
LC_2–2_	298.64	21.53	44.8	3.23	249.4 × 10^3^	0.64	Slightly high; good particle network
LC_2–5_	285.33	18.87	42.8	2.83	225.1 × 10^3^	0.53	Reduced due to latex softening
LC_4–2_	276.45	18.14	41.47	2.72	213.8 × 10^3^	0.49	Stable but lower build-up
LC_4–5_	260.34	16.45	39.05	2.47	194.0 × 10^3^	0.43	Lower thixotropy; smoother flow
LC_6–2_	252.32	14.21	37.85	2.13	181.2 × 10^3^	0.36	Weak structural network
LC_6–5_	245.86	12.89	36.88	1.93	169.9 × 10^3^	0.33	Lowest thixotropy; highly fluid mix

Overall, the decreasing trend of both thixotropic energy and build-up rate from LC_0–0_ to LC_6–5_ clearly indicates that while the incorporation of calcined clay and nano-silica enhances early cohesion and yield stress, excessive polymer content counteracts this effect by promoting lubrication and dispersion of particles. Hence, an optimal balance of limestone, calcined clay, and nano-silica—specifically around LC_2–2_ or LC_4–2_ compositions—offers the best compromise between workability and structural stability. These findings emphasize the critical role of LC3 blend proportions in controlling the time-dependent rheological behavior of sustainable concretes.

### Shrinkage

**Fig. 5:**
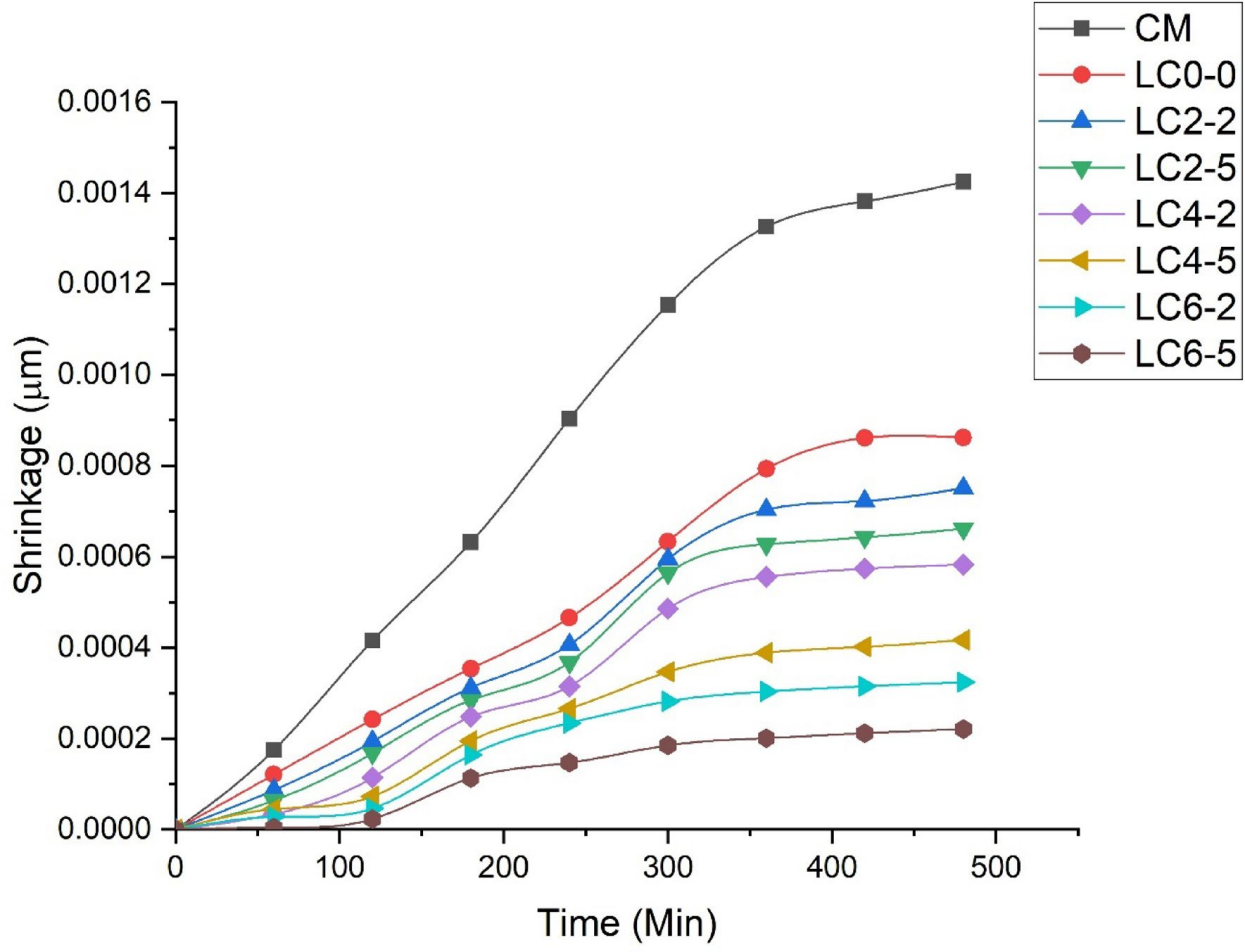
Shrinkage behavior of different LC3 concrete mixes.

In the current research, early-age shrinkage was measured after it was observed that traditional OPC concrete (CM) exhibited a higher shrinkage rate during the first eight hours as shown in Fig. 5. After this period, the shrinkage process slowed down and became nearly constant. Therefore, early-age shrinkage was thoroughly investigated in this experiment. The study also examined how varying amounts of natural rubber latex affected different concrete mixes. When comparing LC3 concrete and traditional OPC concrete (CM) for shrinkage, it was found that LC3 concrete shrank 39.5%less than OPC concrete. Shrinkage reduced by 39% compared to OPC with LC3 alone, and by up to 84% with the inclusion of 6% nano-silica and 5% rubber latex. This lower shrinkage value in LC3 concrete, which is based on calcined clay, can be attributed to two main factors. First, unlike cement, calcined clay did not shrink more. With a particle size smaller than 45 μm, calcined clay had a higher fineness than cement, which prevented bleeding. Second, the limestone powder in LC3 concrete absorbed water during mixing and released it during setting. The water released from lime stone powder compensated the loss of water through evaporation, thus limiting shrinkage. Additionally, it was found that increasing the dosage of nano silica and rubber latex further reduced the shrinkage of LC3 concrete. Nano-silica reacted with free Ca(OH)₂, producing secondary C–S–H gel, refining the pore structure. Rubber latex, upon setting, formed an elastic film that blocked capillary pores, reducing shrinkage and improving stress redistribution under load. After eight hours, the shrinkage reduction in both LC3 concrete was 8% and 45%, respectively, for rubber latex dosages of 2% and 5%. It was observed that rubber latex provided greater resistance to shrinkage compared to nanosilica. Since the unit weight of rubber latex is the same as that of water, it mixed thoroughly with the water and easily penetrated into all the voids in the dry concrete mix during addition. Over time, the rubber latex lost its plasticity and solidified. This solidified material sealed the internal voids of the fresh concrete, effectively resisting further shrinkage. This effect is likely due to the rubber latex preventing water from evaporating from the concrete. Higher dosages of rubber latex were shown to further reduce the shrinkage rate in LC3 concrete.

There are several reasons why the introduction of rubber latex reduced the shrinking properties of LC3 concrete. The initial one is that when the latex of rubber was mixed into traditional LC3 concrete, a tiny film of latex of rubber formed above the water surface, preventing rapid evaporation. This extended the duration of the constant rate period, which referred to the initial stage of drying where water loss occurred. The second one is the addition of rubber latex decreased the surface tension of pore water and increased the viscosity of the pore fluid inside both types of concrete. This leads to reduced moisture saturation, especially at intermediate levels of humidity percentage. This led to a weakening of capillary action, the primary force behind water movement. This disruption of capillary bridges between the concrete mass and the concrete surface lowered the capillary tension within the pore structure, thus slowing down the shrinkage process during evaporation. The enhanced viscosity of the concrete mass further impeded water movement from the interior to the surface, reducing the rate of evaporation in the pre drying hours. The third reason is that limestone powder used in the mix released water up until the cement paste began to set. This water was pulled from the large pores of the limestone powder by capillary suction, which started between the fresh and plastic stages of the concrete. Since the releasing of water from lime stone powder at early age, it helped to delay the development of capillary pressure, which positively influenced the lowering of shrinkage cracking at the plastic state. The additional water provided by the lime stone powder slowed down the formation of capillary pressure in the fresh concrete mass, reduced number of cracks and their width automatically. Lastly, the presence of rubber latex delayed the chemical reaction during the hydration at the initial hardening stage of the concrete due to its organic molecules. Those molecules reduced the polarity of the concrete and increased the specific surface area, thus increasing water demand for hydration. The latex of rubber also reduced the pores in large size and delayed crack initiation, significantly decreasing crack width.

In conclusion, natural rubber latex acted as an effective agent that reduces the shrinkage in concrete. It minimized cracks developed by shrinkage, reduced curling, and joint contraction, and enhanced the performance of concrete. Additionally, the use of nano-silica as a replacement for binder further aided in reducing the shrinkage.

#### Shrinkage strains for the prescribed concrete samples

Four distinct processes were used to compute shrinkage strain in this study: drying of water, capillary action-induced hydrostatic tension, energy on surface of solid gel particles and disjoining pressure.

The shrinkage strain due to drying of water can be evaluated from the present experimental research by.

9 $$\varepsilon = \frac{\Delta V}{V}$$

The strain energy equations of remaining three types suggested by Power of above mechanisms are given below.

The shrinkage strain due to capillary action-induced hydrostatic tension can be expressed as.

10 $$\frac{\Delta V}{V} = \beta \left( {\frac{RT}{{Mv_{w} }} lnh} \right)$$

The shrinkage strain due to energy on surface of solid gel particles can be expressed as.

11 $$\frac{\Delta V}{V} = \frac{2}{3}K\frac{RT}{M}\mathop \smallint \limits_{{h_{1} }}^{{h_{2} }} \frac{{w_{a} }}{{V_{s} }}d\left( {lnh} \right)$$

The shrinkage strain due to disjoining pressure can be expressed as.

12 $$\frac{\Delta V}{V} = \beta f\left( {w_{a} } \right)\frac{RT}{{Mv_{w} }} lnh$$

Where, V = volume of conical concrete sample, M = molecular weight, T = temperature, $w_{a}$ = absorbed water content, V_s_ = absorbed volume, $v_{w}$ = specific volume of water, h = the relative vapor pressure, K = compressible coefficient of solid particles, R = universal gas constant.

Shrinkage strain due to the drying of water was determined experimentally, while the other three types of shrinkage strains were calculated using Power’s equation. The results are presented in Table 4. Shrinkage strain due to drying of water was obtained by taking ratio between shrinkage values of each mix to the total volume of the sample cone.

**Table 4:** Shrinkage strain for both traditional and LC3 concrete from different mechanisms.

Mix	Shrinkage strain			
	Drying of water	Capillary action	Energy on surface	Disjoining pressure
CM	0.001425	0.001211	0.000929	0.001162
LC_0–0_	0.000862	0.000726	0.000678	0.000784
LC_2–2_	0.000751	0.000649	0.000543	0.000679
LC_2–5_	0.000662	0.000547	0.000492	0.000558
LC_4–2_	0.000583	0.000473	0.000378	0.000436
LC_4–5_	0.000417	0.000335	0.000252	0.000325
LC_6–2_	0.000324	0.000257	0.000164	0.000237
LC_6–5_	0.000221	0.000178	0.000951	0.000132

V = Concrete mix volume sample = Volume of conical shrinkage vessel = 285,099 mm^3^.

K = Compressible Coefficient of Solid Particles = 0.85.

R = Universal Gas Constant = 8.314 X 10^3^ N-m-kmol^−1^ k^−1^.

T = Room Temperature = 35 °C.

W_a_ = Absorbed water content = Water added in the mix (from Table 2).

h = Relative Vapour Pressure = 101,325 Pa.

It was discovered that LC3 concrete with 6% nano-silica and 5% rubber latex, which were partially substituted with calcined clay and water, respectively, had an 83% lower shrinkage strain from water evaporation than regular OPC concrete. As the dose of latex of rubber increased, the shrinkage strain for all mechanisms gradually decreased which is shown in Table 4. Shrinkage reduction in LC3 concrete was achieved through a dual mechanism: (i) saturated limestone particles gradually released absorbed water during the plastic stage to counter evaporation-induced stresses, and (ii) rubber latex solidification sealed internal pores during hardening, thereby reducing micro-pore formation and enhancing dimensional stability.

One noteworthy finding is that the shrinkage strain resulting from water evaporation was equal to the total of the shrinkage strains caused by hydrostatic tension, surface energy, and disjoining pressure. This suggested that the overall shrinkage strain was represented by the shrinkage strain resulting from water evaporation. Out of the three methods Power outlined, surface energy-induced shrinking made up more than 60% of the overall contribution.

During the shrinkage period, LC3 concrete specimens without nano silica and rubber latex decreased in weight by 3% more than specimens with nano silica and rubber latex. With very slight variations in the solid components of the pore structure, this suggests that all of the mixes had comparable levels of pore water. In order to prevent water from evaporating from the concrete specimens, a thin layer of rubber latex was produced. On the other hand, the partial replacement of calcined clay with nano silica improved the packing density. By which the void ratio of the concrete mix gradually decreased which helped with the accumulation of water in a little space. Since a small amount of water evaporated, the shrinkage process gradually decreased.

### Mechanical properties

#### Compressive strength

**Fig. 6:**
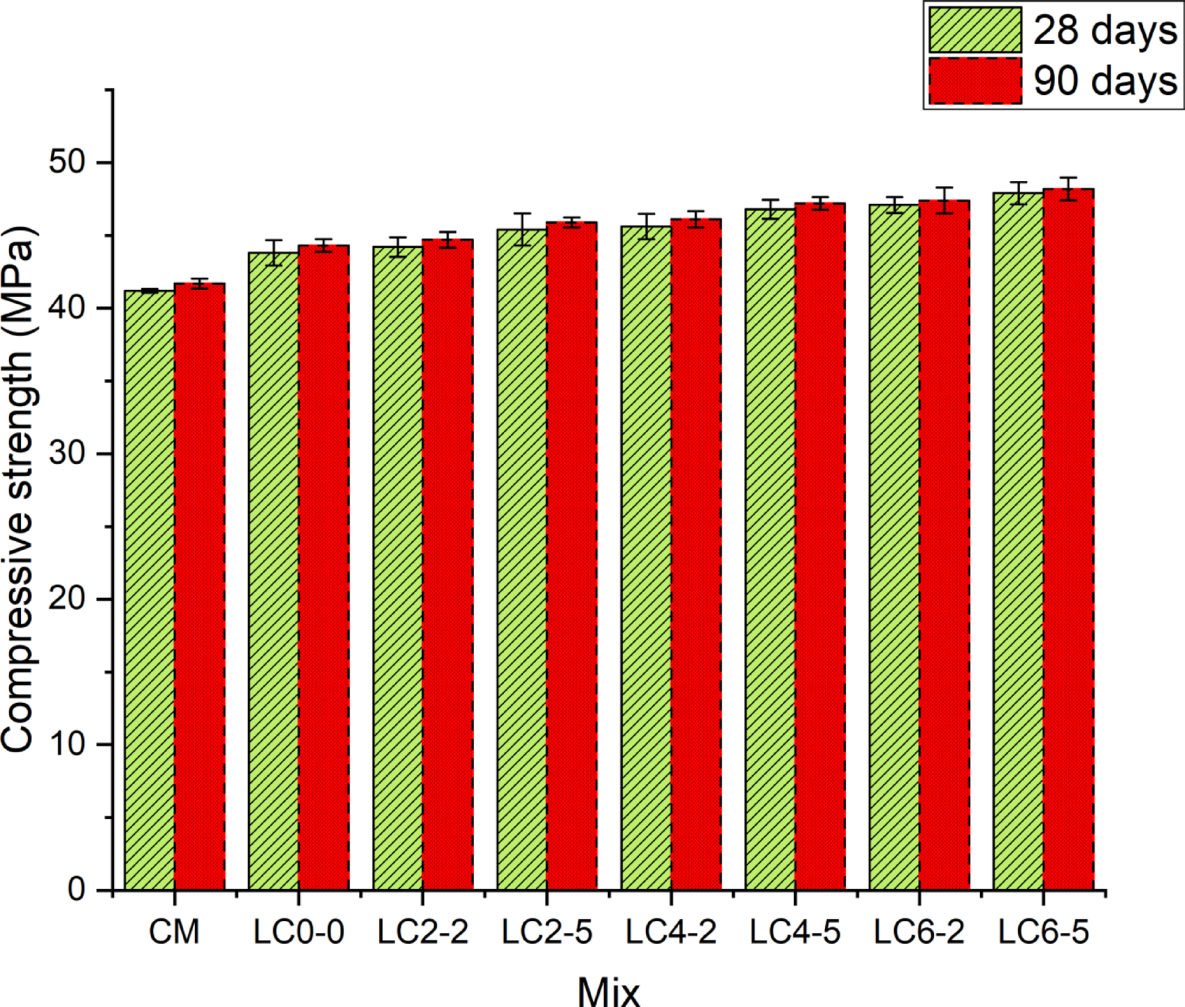
Compressive strength of concrete specimens at 28 days and 90 days.

The Fig. 6 depicts the compressive strength results for the LC3 concrete incorporating nano-silica and rubber latex at different ages. The results indicate that all mixes exhibited an increase in compressive strength over time. The reference mix, conventional mortar (CM), showed the least compressive strength at both 28 and 90 days as compared with all other mixes. The incorporation of limestone calcined cement, nano silica, and rubber latex significantly enhanced the compressive strength, with the extent of improvement dependent on the percentages of nano silica and rubber latex used in the mix. At 28 days, the LC_0–0_ mix showed a slight improvement in compressive strength as compared with CM. This improvement is mainly due to the partial replacement of ordinary cement with limestone calcined cement, which enhances early hydration through the filler effect, increasing matrix density. Concrete samples incorporated with nano silica and rubber latex showed a noticeable increase in compressive strength with different dosages of nanosilica and rubber latex. With a higher proportion of nano silica and rubber latex (LC_6–5_) in concrete specimens, compressive strength specimens showed an 8.8% increase as compared with LC_2–2_. This increase indicates the combined benefits of nano silica’s pozzolanic activity and rubber latex’s flexible bonding effects. Compressive strength results of the 90-day curing period followed a similar trend as that of 28-day results. The 90-day strength reference mix (CM) exhibited almost similar strength as that of the 28-day strength. In contrast, specimens without rubber latex and nano-silica reached a compressive strength of 44.3MPa, showing continuous hydration and filler effects of limestone calcined cement at a higher curing period. An increase in the proportion of nanosilica and rubber latex from 2 to 6% showed the most significant long-term strength enhancement, which signifies the continued pozzolanic reaction of nano-silica with extra available calcium hydroxide and it densifies the concrete matrix due to rubber latex. The improvement in strength can also be attributed to enhanced particle packing density. The ultrafine nano-silica and finely ground limestone fill micro voids between cement and surkhi particles, reducing porosity and leading to a denser matrix. This physical densification complements chemical hydration and improves compressive strength.

The significant improvement in compressive strength with an increase in nano silica and rubber latex is mainly due to synergistic mechanisms. Nano silica contributed to the refinement of the pore structure through its pozzolanic activity, leading to the formation of additional calcium silicate hydrate gel. The enhancement in compressive strength is also linked to a refined interfacial transition zone (ITZ), which is a traditionally weak zone in concrete. Nano-silica fills the microvoids at the ITZ and reduces porosity, while rubber latex forms flexible polymer films that bridge micro-cracks. This dual action improves both strength and ductility, contributing to more uniform stress distribution under load. This refinement fills the capillary pores in addition to the improvement of the interfacial transition zone (ITZ) between the cement paste and aggregates. On the other hand, rubber latex incorporation into concrete samples improves the bonding within the cementitious matrix by enhancing the flexibility and reducing the micro-crack propagation and it increases the durability and load-bearing capacity of the concrete. Later age strength results showed a more pronounced increase in strength compared to 28-day strength results for the concrete specimens containing a higher proportion of nano silica and rubber latex. The LC_6–5_ concrete specimens at 90 days showed a 15.6% increase in compressive strength as compared with conventional mortar. The significant improvement shows the combined benefits of nano silica and rubber latex utilization in improving the mechanical characteristics of concrete. The integration of nano silica and rubber latex into LC3 concrete enhances the compressive strength due to the combined effects of the improved concrete density matrix, pozzolanic reaction, and better microstructure formation and it makes the LC_6–5_ concrete samples, the most promising mix for structural applications with high strength. Also, the strength development kinetics of LC3 concrete with nano-silica and rubber latex indicate accelerated early-age hydration, likely due to the nucleation effect of nano-silica. However, the 28-to-90-day strength gain in LC3 mixes (e.g., LC6-5) remained higher than that of OPC concrete, showing sustained long-term pozzolanic activity. This suggests that nano-silica not only accelerates hydration but also contributes to long-term strength through continued secondary C–S–H formation. Similar findings were reported by prior research, where incorporating 2% nano-silica and 12.5% metakaolin increased compressive strength by up to 35%, mainly due to enhanced C–S–H gel formation and denser matrix packing^46^. Another study showed that substituting silica fume with 1–5% nano-silica in self-compacting concrete improved compressive strength by refining pore structure and promoting pozzolanic reactions^47^. Likewise, adding 3% nano-silica to barite aggregate concrete resulted in an 18% increase in compressive strength, attributed to reduced porosity and improved interfacial transition zone (ITZ)^48^. Interestingly, the mixes that showed reduced yield stress and viscosity due to rubber latex and nano-silica incorporation also exhibited higher compressive strengths. This suggests that improved dispersion and packing during the fresh stage contributed to a denser and more homogeneous microstructure upon hardening, which correlates well with increased load-bearing capacity. Increased compressive strength in LC3 concretes with nano-silica and rubber latex is also indicative of improved durability. Denser microstructure and lower microcracking potential reduce permeability and shrinkage-induced degradation, suggesting these mixes are not only stronger but also more resilient to environmental exposure. The current study aligns with these findings, as nano-silica reacts with free Ca(OH)₂ to form additional C–S–H gel, densifying the matrix. Rubber latex further enhances strength by sealing pores and reducing shrinkage-induced cracking. Achieving higher compressive strength with reduced clinker content (33% in LC3) underscores the sustainability advantage of this approach. The synergistic use of SCMs like surkhi and limestone, along with performance-enhancing nano-additives, allows for strength gains without compromising environmental goals. This synergy leads to higher compressive strength, especially at optimum nano-silica and rubber latex dosages.

#### Split tensile strength

**Fig. 7:**
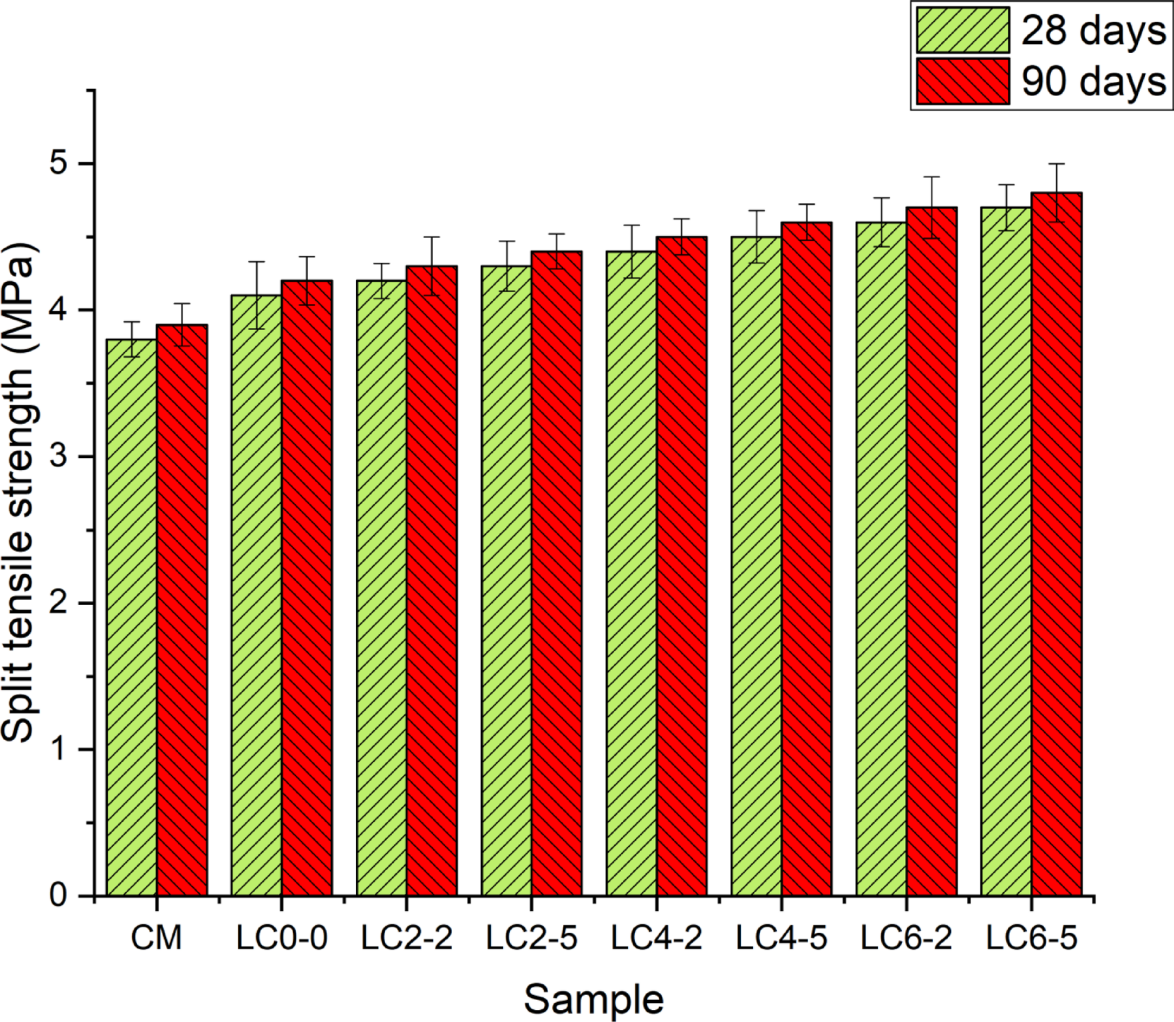
Split tensile strength of concrete specimens at 28 days and 90 days.

The split tensile strength results for LC3 concrete incorporated with nano silica and rubber later at 28 days and 90 days are illustrated in Fig. 7. The results show that all specimens exhibit an increase in tensile strength over time. The tensile strength of specimens almost followed a similar trend as that of compressive strength. For reference mix, conventional mortar showed the least tensile strength at 28 and 90 days, and the limestone calcined cement, nano silica, and rubber latex-based specimens showed higher tensile strength as compared with conventional mortar. At 28 days, conventional mortar exhibited a split tensile strength of 3.8 MPa, whereas, limestone calcined cement without nano silica and rubber latex-based concrete specimens (LC_0–0_) showed marginal improvement in split tensile strength of about 7.8%. Nano-silica and fine limestone particles filled voids between larger cement grains, optimizing particle packing and reducing pore connectivity. This densified the matrix and enhanced both mechanical strength and shrinkage resistance. A higher proportion incorporation of rubber latex and nano-silica increases the tensile strength steadily from 4.2 MPa for LC_2–2_ species to 4.7 MPa for LC_6–5_. This shows that the incorporation of nano silica and rubber latex has a positive effect on split tensile strength positively at early ages by improving the bond strength within the concrete matrix and reducing the micro-cracks. Rubber latex acts as a microcrack arrestor by forming continuous polymeric films throughout the matrix. These films provide a ductile phase that can deform under tensile loading, delaying crack initiation and propagation. This mechanism not only improves tensile strength but also enhances the post-cracking energy absorption, contributing to the concrete’s ductile response. At a higher curing period, the tensile strength of CM increased from 3.8 to 3.9 MPa, whereas LC_0–0_ concrete specimens reached a maximum tensile strength of 4.2 MPa. This shows the continuous hydration and improved matrix density of limestone calcined cement-based concrete. The enhanced split tensile strength can be partially attributed to the refinement of the interfacial transition zone (ITZ). Nano-silica densifies the ITZ by filling microvoids and reacting with portlandite, while rubber latex improves the mechanical interlocking and bonding through flexible film formation. This refined ITZ enhances stress transfer from the matrix to the aggregates, thereby improving tensile resistance.

The LC_2–2_ and LC_6–5_ specimens show tensile strength of 4.3 MPa and 4.8 MPa and it is a 10% and 23% increase in tensile strength than reference mix respectively. A higher proportion of nano-silica and rubber latex provided the most significant long-term strength enhancement, indicating the continuous progress of pozzolanic activity by rubber latex and nano-silica, directly contributing to the enhancement of tensile properties over time. Rubber latex incorporation into concrete improves the bond between the concrete matrix and reduces the crack propagation potential under tensile loading. The combination of nano silica and rubber latex provides a more resilient and homogenous matrix, that withstands tensile stresses effectively. The increase in tensile strength of concrete specimens from 28 to 90 days was more significant for mixes with a high proportion of latex and nano-silica, indicating the benefits of these materials in long-term tensile strength development. LC6-5 specimens exhibited a 90-day tensile strength of 4.8 MPa which is 14.2% higher strength than LC_0–0_ specimens. This enhancement of strength shows the significant role of nano silica and rubber latex in improving the tensile characteristics of LC3 concrete by promoting denser and more flexible microstructure. The ratio of split tensile strength to compressive strength remained within the typical range of 8–12% across all mixes, but with slightly higher values in mixes containing rubber latex and nano-silica. This suggests that the improvements in microstructure and crack-bridging mechanisms disproportionately favor tensile performance, which is typically more sensitive to microcrack propagation and matrix homogeneity. Previous studies found that adding 2% nano-silica and 12.5% metakaolin enhanced split tensile strength by up to 27%, attributed to the formation of additional C–S–H gel and improved bonding at the aggregate-paste interface^46^. Similarly, replacing silica fume with 1–5% nano-silica in self-compacting concrete showed higher tensile strength, linked to better particle packing and reduced microcracks^47^. Research on barite aggregate concrete incorporating 3% nano-silica also reported improved tensile strength due to a denser ITZ^48^. In the current study, nano-silica contributed to pozzolanic reactions that refined pore structure, while rubber latex formed a flexible film that bridged microcracks and enhanced tensile load transfer. This synergistic action reduced crack propagation and improved tensile strength. The observed strength increase aligns with prior work, confirming the effectiveness of optimized nano-silica and rubber latex content. Improved split tensile strength in LC3 concretes is particularly advantageous for structural members subjected to indirect tension such as pavements, slabs, tunnel linings, and composite elements where crack control is critical. The synergistic effects of nano-silica and rubber latex could reduce the need for excessive reinforcement or crack-control measures in such applications.

## Microstructure analysis

### FTIR Test

**Fig. 8:**
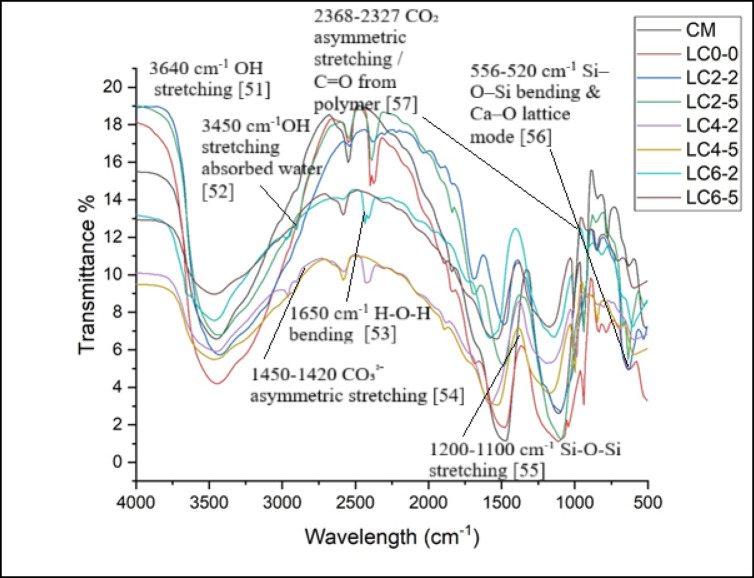
FTIR test results for different concrete samples.

Fourier Transform Infrared Spectroscopy (FTIR) is widely employed to investigate the molecular structure and chemical bonding of hydration products and carbonation phases. To clearly detect bond breakage in CaCO₃, Ca(OH)₂, and C–S–H phases using FTIR, it is essential to understand their characteristic absorption bands and to interpret changes such as peak shifts, broadening, intensity reductions, or peak disappearance as indicators of bond rupture or phase transformation. The FTIR results for 28 days of curing of LC3 concrete for substituting calcined clay up to 6% and water up to 5% by nano silica and rubber latex simultaneously are displayed in Fig. 8. The stretching vibration of LC3 concrete was recorded at 1062 cm^−1^, which was shifting towards low wave numbers like 940 cm^-1^, 894 cm^-1^, 845 cm^−1^, 798 cm^−1^, 771 cm^−1^, 764 cm^−1^, 755 cm^−1^ against the mixes LC_2–2_, LC_2–5_, LC_4–2_, LC_4–5_, LC_6–2_ and LC_6–5_ respectively with respect LC3 concrete without any replacement. There was an approximate shift of 46 cm^−1^, 95 cm^−1^, 77 cm^−1^, 142 cm^−1^, 169 cm^−1^, 176 cm^−1^ and 185 cm^−1^. According to the results, an increased amount of both nano silica and rubber latex in LC3 concrete produced the extra C–S–H gel by reducing the sufficient amount of porosity by the rubber latex, which alerted the LC3 concrete and improved the strength parameters. Typical FTIR bands observed in cementitious materials during the C–S–H gel formation included Si–O–Si asymmetric stretching vibrations was appearing around 950–1100 cm⁻^1^, Si–O bending vibrations appeared near 450–500 cm⁻^1^, OH stretching formed absorbed water around 3450 cm⁻^1^, and H–O–H bending vibrations was found approximately at 1630 cm⁻^1^. The reduction of 950–1100 cm⁻^1^ indicated the breakdown of Si–O network. The loss of 3450 & 1630 cm⁻^1^ peaks suggests about the dehydration of C–S–H gel.

Large bands around 1613–1667 cm^−1^ are H–O–H stretching vibrations, while bands like 1333–1426 cm^−1^ are OH groups of bending vibrations of products of the hydrated reaction of water^49^. These bands in LC3 concrete demonstrated the reaction of Ca(OH)_2_ with nano silica particles. The O–H stretching was found at 3640 cm^−1^, whereas the OH bending was found at 520 cm⁻^1^. The Si–O stretching and bending vibrations of silicate were found to be the vibrational bands for the concrete at 556 and 986 cm^−1^, respectively. Strong bands in the 1100–1200 cm^−1^ range were caused by the presence of nano silica stretching vibrations. Additionally, the presence of rubber latex was responsible for the band in the 2327–2368 cm^−1^ range^50^. The disappearance of 3640 cm⁻^1^ band indicated the dihydroxylation whereas the reduction in 520 cm^−1^ confirmed about the Ca–O lattice disruption. Water was the cause of the strong bands at about 1300–1450 cm^−1^. Again, the CaO stretching vibration of CaCO_3_ was the cause of the tentative band that appeared in the 2200–2350 cm^−1^ range.

### Thermogravimetry analysis

**Fig. 9:**
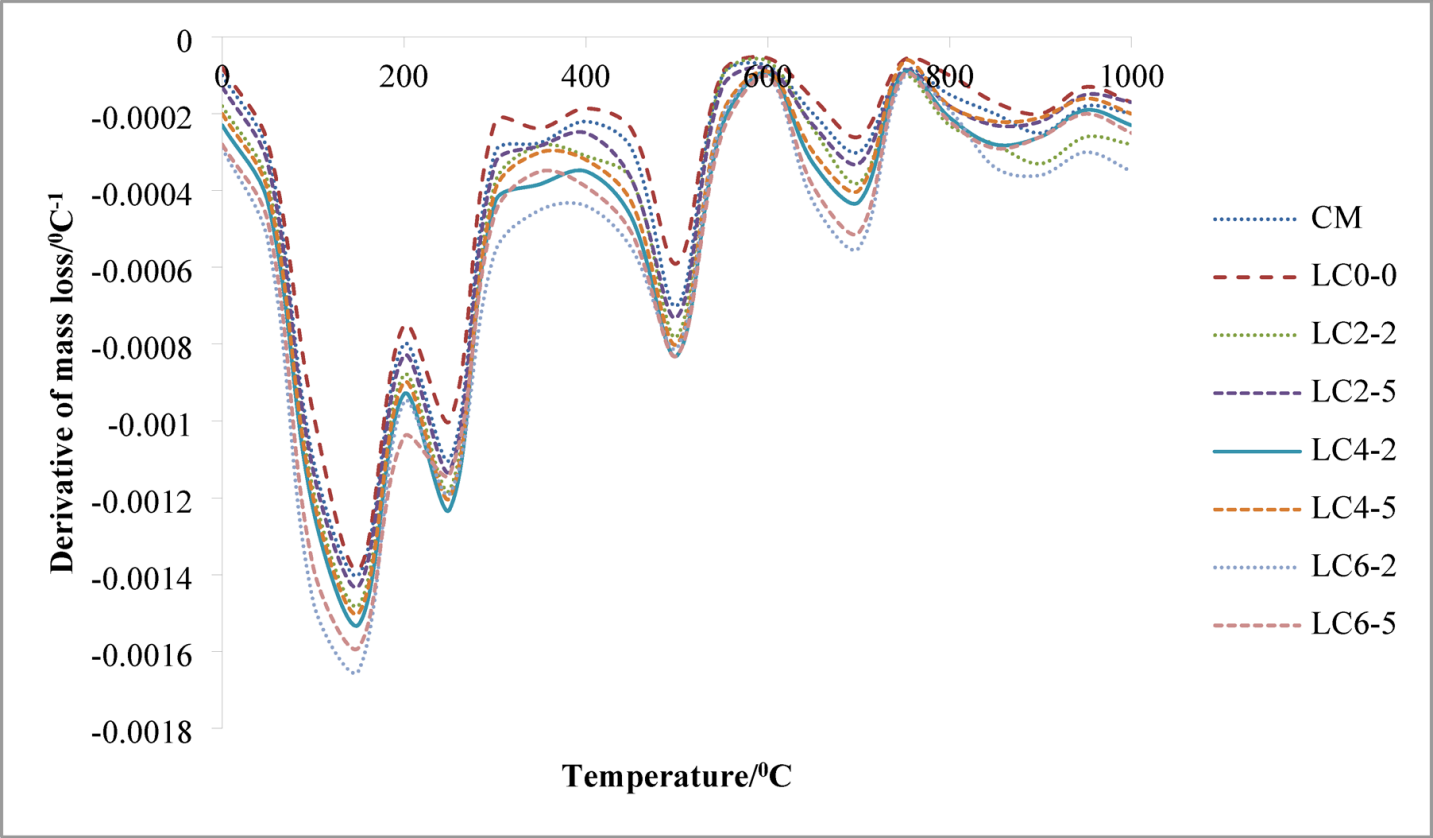
DTG curves for various mixes of concrete.

**Fig. 10:**
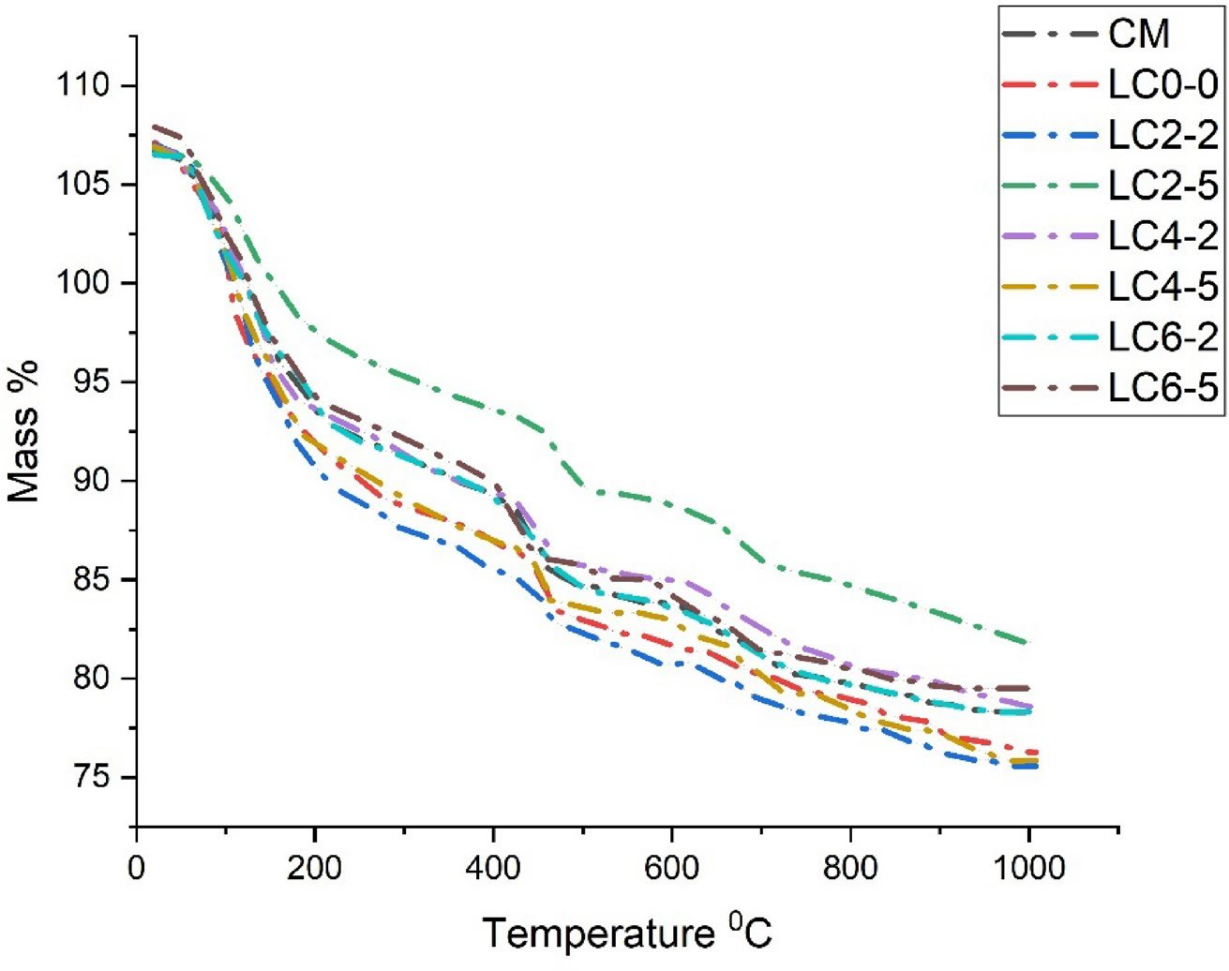
Thermo gravimetric curves for different mixes of concrete.

Thermogravimetry Analysis (TGA) findings for traditional OPC concrete and LC3 concrete cured for the 28-day water curing. To observe each phase, the results were plotted using derivative thermogravimetric (DTG), as seen in Fig. 9. For all concrete samples, the primary mass loss transition took place across three temperature ranges. The loss of water at various hydrate phases, including CSH gel, ettringite, and gehlenite (C_2_ASH_8_), was the primary cause of the first mass loss up to 300^0^C. Dehydroxylation of calcium hydroxide [Ca(OH)_2_] was the cause of the second major mass loss between 300 and 550 °C^44^. Decarbonation of calcium carbonate (CaCO_3_) made up the third major mass loss between 500 and 1,000^0^C^45^. The decarbonization of calcium carbonate (CaCO_3_) was the third major mass loss for temperatures between 550 and 1000 °C. All findings for DTG of traditional OPC concrete samples on the completion of 28 days revealed the phases as follows: ettringite between 75 and 85 °C, C–S–H between 155 and 170 °C, C_2_ASH_8_ between 155 and 165 °C, and Ca(OH)_2_ between 455 and 465 °C. The CaCO_3_ showed two prominent peaks at 750 and 950 °C. The decomposition of regular calcite produced the first CaCO_3_ peak, and variations in the particle size, surface area, CO_2_ partial pressure, and contaminants like magnesium that impacted calcite breakdown are likely to have caused the second peak to vary. For the LC3 concrete mix, it was observed that the peak of C–S–H curves increased with increasing the dose of nano-silica content due to the higher pozzolanic reaction rate when nano-silica was added. The loss of mass for LC3 concrete after curing for 28 days is shown in Table 5. The mass loss of the C–S–H + C_2_ASH_8_ + ettringite phases rose as the percentage of nano silica content rose. As compared to traditional OPC concrete, the mass loss for LC3 was greater. With increased nano-silica content, the mass loss associated with Ca(OH)₂ decomposition between 400–550 °C decreased, indicating enhanced pozzolanic reaction where nano-silica consumed free Ca(OH)₂ to form additional –S–H, thereby improving microstructure. This occurred as a result of the percentage of nano silica increasing the pozzolanic activity of LC3 concrete. Because Ca(OH)_2_ was consumed by nano-silica in the pozzolanic reaction, the mass loss of the Ca(OH)_2_ phase of concrete tended to decrease when nano-silica was introduced as a replacement material for calcined clay. The loss of mass versus temperature is shown in Fig. 10. The mass loss of CaCO_3_ for controlled OPC concrete was higher than the LC3 concrete because the controlled concrete samples were directly exposed to carbonation due to the formation of weaker ITZ. OPC concrete sample had larger pores and more linked channels, which led to greater CO_2_ diffusion and higher carbonation. Since rubber latex and secondary C–S–H gel formed by nano-silica sealed the capillary pores and increased the quality of the ITZ layer, LC3 concrete was affected by the carbonation at a negligible amount.

**Table 5:** Loss of mass of OPC concrete and LC3 concrete cured at 28 days.

Mix	Mass loss %			Temperature (^o^C)		
	CSH + C_2_ASH_8_ + Ettringite	Ca(OH)_2_	CaCO_3_	CSH + C_2_ASH_8_ + Ettringite	Ca(OH)_2_	CaCO_3_
CM	9.267	4.385	5.623	$\sim 300^{0} C$	$\sim 455^{0} C - 465^{0} C$	$\sim 750^{0} C \& \sim 950^{0} C$
LC_0–0_	10.329	5.369	6.984	$\sim 310^{0} C$	$\sim 460^{0} C - 470^{0} C$	$\sim 750^{0} C \& \sim 950^{0} C$
LC_2–2_	12.537	7.496	8.279	$\sim 315^{0} C$	$\sim 450^{0} C - 460^{0} C$	$\sim 750^{0} C \& \sim 950^{0} C$
LC_2–5_	15.348	9.384	10.643	$\sim 320^{0} C$	$\sim 445^{0} C - 455^{0} C$	$\sim 750^{0} C \& \sim 950^{0} C$
LC_4–2_	14.462	8.523	9.826	$\sim 320^{0} C$	$\sim 440^{0} C - 450^{0} C$	$\sim 750^{0} C \& \sim 950^{0} C$
LC_4–5_	17.354	10.782	11.716	$\sim 325^{0} C$	$\sim 435^{0} C - 445^{0} C$	$\sim 750^{0} C \& \sim 950^{0} C$
LC_6–2_	16.852	9.418	10.275	$\sim 330^{0} C$	$\sim 430^{0} C - 440^{0} C$	$\sim 750^{0} C \& \sim 950^{0} C$
LC_6–5_	18.945	11.327	12.538	$\sim 335^{0} C$	$\sim 425^{0} C - 435^{0} C$	$\sim 750^{0} C \& \sim 950^{0} C$

In summary, The thermogravimetric mass losses can be explicitly correlated with distinct phase decompositions. The first major loss, observed below ~ 300 °C, is attributed to the dehydration of C-S–H gel, ettringite, and C₂ASH₈. Between 400 and 550 °C, the mass loss corresponds to the dehydroxylation of Ca(OH)₂. The third significant loss, occurring between ~ 550 °C and 1000 °C, is due to CaCO₃ decarbonation. Notably, CaCO₃ displays two decomposition peaks: the first around 750 °C, representing regular calcite, and the second near 950 °C, likely influenced by variations in particle morphology, surface area, partial CO₂ pressure, and presence of impurities like magnesium.

Again from Table-5, it has been found that as the nano-silica content increases in the mixes (LC_2–2_, LC_2–5_, LC_4–2_, LC_4–5_, LC_6–2_, LC_6–5_), the mass loss attributed to Ca(OH)₂ decreases, while the mass loss from C–S–H, C₂ASH₈, and ettringite formation increases. Nano-silica (SiO₂) is highly reactive due to its extremely fine particle size and high surface area. It reacts with calcium hydroxide [Ca(OH)₂], a by-product of cement hydration, in the following pozzolanic reaction.

13 $$SiO_{2} + Ca\left( {OH} \right)_{2} + H_{2} O \to CSH$$

For example, mix CM (control) had shown 4.385% Ca(OH)₂, while LC_6–5_ (with higher nano-silica) had shown a lower 11.327% Ca(OH)₂ despite having the highest C–S–H-related mass loss (18.945%). This suggests that Ca(OH)₂ was being actively consumed to form more C–S–H, which was the primary strength-giving phase in concrete. Again, Increased formation of C–S–H improved the matrix densification, reducing porosity and increasing compressive strength. The data thus supported a quantitative correlation: as Ca(OH)₂ was consumed by nano-silica, C–S–H content rises, directly contributing to strength gains.

The quantitative relationship between Ca(OH)₂ consumption and the generation of additional hydration products can be clearly observed from Table 5. In the control mix (CM), the mass loss associated with Ca(OH)₂ is 4.385%, while the mass loss for the combined C–S–H, ettringite, and C₂ASH₈ phases is 9.267%. As nano-silica is introduced into the LC3 mixes, both the Ca(OH)₂ and C–S–H-associated mass losses increase significantly, indicating progressive pozzolanic activity. For example, in mix LC_2–2_, the Ca(OH)₂ mass loss increases to 7.496%, with a corresponding C–S–H-associated loss of 12.537%. This trend continues with mix LC_4–5_, where Ca(OH)₂ mass loss reaches 10.782% and the C–S–H-associated phase loss rises to 17.354%, suggesting a peak in pozzolanic reactivity. The highest values are observed in mix LC_6–5_, with 11.327% Ca(OH)₂ loss and 18.945% for C–S–H-related products. This progression highlights a direct correlation between Ca(OH)₂ consumption and the formation of strength-contributing phases. As nano-silica content increases, more Ca(OH)₂ is consumed in pozzolanic reactions, resulting in the generation of greater amounts of C–S–H gel, which densifies the microstructure and is directly responsible for improved mechanical strength in LC3 concrete systems.

## Conclusions

This study aimed to evaluate the influence of nano-silica and rubber latex on the rheological, shrinkage, mechanical, and microstructural properties of LC3 concrete in comparison to conventional OPC concrete. Based on the comparative analysis, the following conclusions were drawn.

(i) Rheological Behavior: Moderate variation in flowability was observed between OPC and LC3 concretes due to differences in water demand and amorphous phase content. The presence of finely ground surkhi in LC3 concrete led to increases of 3% and 5% in static and dynamic yield stress, respectively, relative to traditional OPC concrete.
(ii) Effect of Nano-Silica and Rubber Latex on Rheology: The incorporation of nano-silica (up to 6% replacing surkhi) and rubber latex (up to 5% replacing water) significantly influenced the rheological behavior of LC3 concrete. Notable reductions were observed in static yield stress (15%), relative viscosity (35.42%), structural built up rate (43.1%) and thixotropic energy (27.8%). These improvements were attributed to the finer particle packing and improved dispersion resulting from the synergistic effect of nano-silica and rubber latex.
(iii) Volumetric Shrinkage: LC3 concrete exhibited 39% lower shrinkage than OPC concrete, likely due to the water-retentive nature of limestone powder. With further replacement by nano-silica and rubber latex, shrinkage reduced by up to 84%, primarily due to increased particle packing density and void filling by rubber latex, which transitions from liquid to solid during setting, effectively sealing capillary pores.
(iv) Mechanical Strength: Both compressive and split tensile strengths of LC3 concrete—regardless of additive content—consistently exceeded those of traditional OPC concrete. The incorporation of nano-silica enhanced the packing density, contributing significantly to LC3’s classification as a high-performance concrete.
(v) FTIR Analysis: With increased nano-silica and rubber latex content, peak intensities corresponding to CaCO₃ and Ca(OH)₂ increased in LC3 concrete. The SiO₂ peak was more prominent in LC3 samples than in OPC concrete, indicating higher silicate presence and enhanced pozzolanic activity.
(vi) Thermogravimetric Analysis (TGA): LC3 concrete with higher nano-silica and rubber latex content exhibited greater mass loss associated with C–S–H, C₂ASH₈, and ettringite phases, suggesting their higher formation. Concurrently, a reduction in Ca(OH)₂ content was observed due to its consumption by nano-silica in the pozzolanic reaction.
(vii) Environmental Implications: The proposed LC3 formulation presents a viable strategy for reducing the carbon footprint of concrete through lower clinker usage. The use of alternative, locally available materials like surkhi, rubber latex, and nano-silica supports the transition to more environmentally responsible construction practices.
(viii) Rubber Latex as a Sustainable Admixture: Due to its workability-enhancing properties, organic nature, and environmental safety, rubber latex shows promise as a sustainable superplasticizer. Its increased utilization may stimulate natural rubber cultivation, contributing to both ecological and economic sustainability.
(ix) Recommendations for Further Research: Future investigations should explore higher dosages of rubber latex at reduced water-to-cement ratios to further enhance microstructural and mechanical performance. Additionally, the combined use of rubber latex with sustainable fibers and SCMs in strain-softening LC3 systems merits detailed evaluation.

In conclusion, the integration of nano-silica and rubber latex in LC3 concrete not only improves performance across multiple dimensions but also aligns with the global objectives of sustainable construction. Future studies should focus on long-term durability, field performance, and cost-benefit analyses to support large-scale adoption.

## Data Availability

The datasets during and/or analyzed during the current study are available from the corresponding author upon reasonable request.

## Abbreviations

LC3: Limestone calcined clay cement
OPC: Ordinary Portland cement
C-S-H: Calcium silicate hydrate gel
TGA: Thermo gravimetric analysis
FTIR: Fourier transform infrared spectroscopy
SSD: Saturated surface dry
